# Combinatorial Analysis of miRNAs and tRNA Fragments as Potential Biomarkers for Cancer Patients in Liquid Biopsies

**DOI:** 10.3390/ncrna11010017

**Published:** 2025-02-14

**Authors:** Ilias Glogovitis, Silvia D’Ambrosi, Mafalda Antunes-Ferreira, Monica Chiogna, Galina Yahubyan, Vesselin Baev, Thomas Wurdinger, Danijela Koppers-Lalic

**Affiliations:** 1 Department of Neurosurgery, Cancer Center Amsterdam, Amsterdam University Medical Centers, 1081 HV Amsterdam, The Netherlands; ilias@uni-plovdiv.bg (I.G.); s.dambrosi@amsterdamumc.nl (S.D.); m.antunesferreira@amsterdamumc.nl (M.A.-F.);; 2 Department of Molecular Biology, University of Plovdiv, 4000 Plovdiv, Bulgaria; gyahubyan@uni-plovdiv.bg (G.Y.); baev@uni-plovdiv.bg (V.B.); 3 Department of Statistical Sciences “Paolo Fortunati”, University of Bologna, 40126 Bologna, Italy; monica.chiogna2@unibo.it; 4 Leiden University Medical Center, Mathematical Institute, Leiden University, 2333 CA Leiden, The Netherlands

**Keywords:** miRNAs, isoforms, tRNAs, liquid biopsy, colon cancer, prostate cancer, bioinformatics

## Abstract

**Background:** Liquid biopsy has gained significant attention as a non-invasive method for cancer detection and monitoring. IsomiRs and tRNA-derived fragments (tRFs) are small non-coding RNAs that arise from non-canonical microRNA (miRNAs) processing and the cleavage of tRNAs, respectively. These small non-coding RNAs have emerged as pro-mising cancer biomarkers, and their distinct expression patterns highlight the need for further exploration of their roles in cancer research. **Methods:** In this study, we investigated the differential expression profiles of miRNAs, isomiRs, and tRFs in plasma extracellular vesicles (EVs) from colorectal and prostate cancer patients compared to healthy controls. Subsequently, a combinatorial analysis using the CombiROC package was performed to identify a panel of biomarkers with optimal diagnostic accuracy. **Results:** Our results demonstrate that a combination of miRNAs, isomiRs, and tRFs can effectively di- stinguish cancer patients from healthy controls, achieving accuracy and an area under the curve (AUC) of approximately 80%. **Conclusions:** These findings highlight the potential of a combinatorial approach to small RNA analysis in liquid biopsies for improved cancer diagnosis and management.

## 1. Introduction

Cancer remains a leading cause of mortality worldwide, emphasizing the need for effective early detection and monitoring strategies. Liquid biopsies have revolutionized cancer diagnosis and monitoring by offering a non-invasive alternative to traditional tissue biopsies. The assays relying on analyzing various biomolecules released by tumor cells into bodily fluids, such as blood, urine, and cerebrospinal fluid, have emerged as pro-missing tools in cancer research and clinical practice [1]. MicroRNAs (miRNAs) and transfer RNAs (tRNAs) are two fundamental classes of non-coding RNAs (ncRNAs) with diverse roles in cellular biology. MiRNAs are small regulatory RNAs known for their abi- lity to post-transcriptionally modulate gene expression, while tRNAs are key players in protein synthesis [2,3]. Recently, microRNAs and tRNAs have attracted attention as potential biomarkers in liquid biopsies due to their involvement in multiple aspects of cancer biology [4].

miRNAs are short, single-stranded RNA molecules, approximately 21–25 nucleotides in length, that play a pivotal role in regulating gene expression. They act as post-transcriptional regulators by binding to the 3′ untranslated region (UTR) of target messenger RNAs (mRNAs), leading to mRNA degradation or translational inhibition, impacting various cellular processes [5,6]. The dysregulation of miRNAs is a hallmark of cancer and is associated with tumor initiation, progression, and metastasis [6,7]. They are known for their stability in various bodily fluids, making them ideal candidates for liquid biopsy-based assays [5,8]. Detection methods such as reverse transcription-polymerase chain reaction (RT-PCR) and next-generation sequencing (NGS) have enabled the identification of specific miRNA signatures for different cancer types, providing valuable diagnostic and prognostic information [5,6]. Recent advancements in sequencing technologies have demonstrated that miRNA genes can produce a variety of sequence variants, diverging from canonical miRNAs. These variants, known as isomiRs, can differ in sequence length (templated isomiRs), end modifications (non-templated isomiRs), or nucleotide composition (polymorphic isomiRs). They arise from variations in the canonical biogenesis pathway or non-canonical pathways [2,9,10,11]. IsomiRs can add a layer of complexity to liquid biopsy analyses [7]. These isoforms exhibit tissue- and disease-specific expression patterns, further enhancing the diagnostic potential of miRNAs in liquid biopsies [8]. Understanding the functional implications of miRNA isoforms in cancer biology is an evolving area of interest.

tRNAs, on the other hand, are essential for protein synthesis, as they deliver amino acids to the ribosome during translation, but emerging evidence suggests their involvement in non-canonical functions. tRNAs and tRNA-derived fragments (tRFs) have been linked to cellular responses to oxidative stress [12,13] and cancer [14,15,16]. tRFs are small non-coding RNAs generated through specific cleavage of tRNAs and involved in various biological processes [17,18,19]. The most frequently investigated tRFs are 5′-tRFs and 3′-tRFs, which are generated from the 5′ and 3′ ends of mature tRNAs, respectively. Additionally, tRFs can also originate from the internal regions of tRNAs, known as internal tRNA-derived fragments (i-tRFs) [20,21]. Aberrant tRNA expression and the presence of tRFs have been observed in various cancer types [22,23]. These molecules are detectable in bodily fluids, providing potential biomarkers for cancer diagnosis and monitoring [24,25].

Although miRNAs and tRNAs have been extensively studied as cancer biomarkers in tissue biopsies, which are associated with risks and limitations, the potential of isomiRs and tRFs in liquid biopsies remains largely unexplored. These small ncRNAs can offer unique advantages due to their tissue-specific expression patterns and stability in biolo-gical fluids [26,27]. IsomiRs are not merely artifacts, they actively participate in gene re-gulation by interacting with the RNA-induced silencing complex (RISC) and shifting the targetome due to alterations in their seed sequences, potentially leading to divergent biological effects [28]. Similarly, tRFs have been implicated in various cellular processes, including gene regulation and stress responses [20,29]. In the present study, we focused on the expression profiles of microRNAs, isomiRs, and tRFs of plasma extracellular vesicles (EVs) from colorectal and prostate cancer. RNA expression data were retrieved from the Extracellular Vesicles database (EVAtlas) [30]. EVAtlas is an open-access repository re-presenting an ideal framework because it makes available ncRNA-sequencing profiles from 24 conditions, 8 liquid sources, and 7 ncRNA types. We employed CombiROC-based combinatorial analysis of miRNAs and isomiRs (from now on—miRNAs/isomiRs) and tRFs to identify the optimal combinations of these small RNAs as potential biomarkers in two types of cancer. The application of this innovative approach to small RNA analysis in liquid biopsies, specifically EVs, has great promise for cancer diagnosis and treatment. Addressing existing knowledge gaps and advancing the field of liquid biopsy-based cancer diagnostics can lead to better patient outcomes and support the development of personalized medicine.

## 2. Results

**Figure 1:**
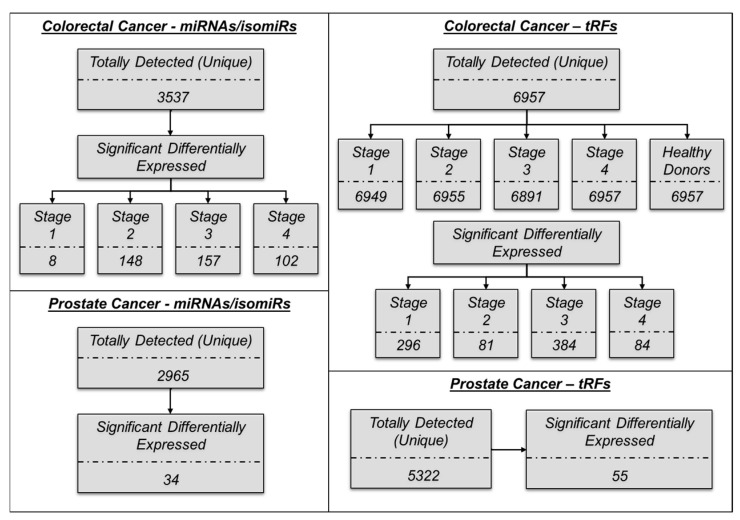
Summary of the analysis findings.

**Figure 2:**
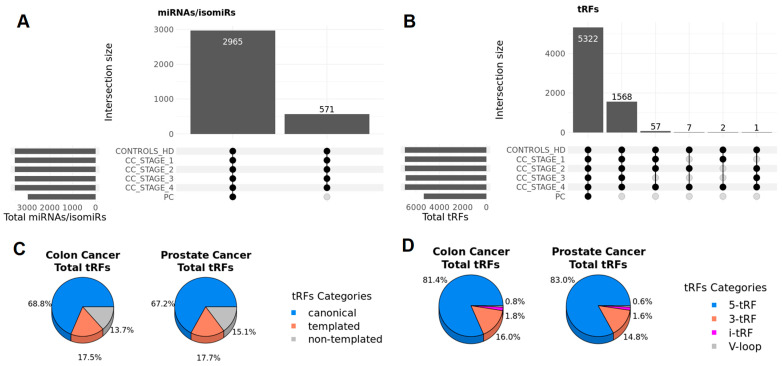
Distribution of total miRNAs/isomiRs and tRFs in colon cancer and prostate cancer. Intersection of miRNAs/isomiRs (**A**) and tRFs (**B**) across colon cancer and prostate cancer groups and healthy donors. Distribution of total detected miRNA/isomiRs (**C**) and tRF types (**D**) per stage in colon cancer and prostate cancer.

**Figure 3:**
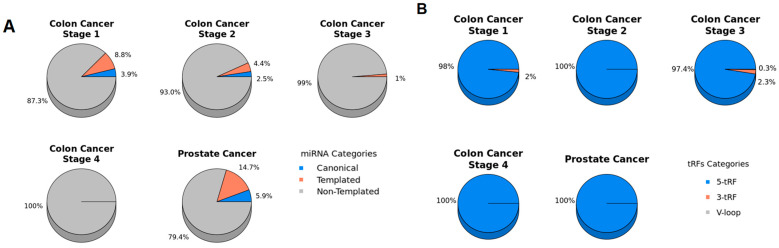
Distribution of the differentially expressed miRNAs/isomiR and tRF types in colon cancer and prostate cancer. (**A**) miRNA and isomiRs (**B**) tRF types detected per stage in colon cancer and prostate cancer.

Due to the tissue-specific and cancer-determined expression patterns of miRNA/isomiRs and tRDs, the first step in our analysis was to identify all types of small ncRNAs in samples from the four stages of colon cancer, as well as prostate cancer. We identified a total of 3536 miRNAs/isomiRs and 6957 tRFs in colon cancer samples and 2965 miRNAs/isomiRs and 5322 tRFs in prostate cancer (Figure 1 and Figure 2). To discover a circulating miRNA and tRF signature for monitoring purposes, we hypothesized that candidate biomarkers would be significantly differentially expressed in cancer samples compared to healthy control samples. Notably, the significantly differentially expressed miRNA-derived sequences were predominantly isomiRs rather than canonical miRNAs. This finding underscores the importance of considering isomiRs in biomarker discovery, as they may play unique roles in cancer biology and offer novel insights into disease mechanisms (Figure 2A,C and Figure 3A).

### 2.1. Discovery of the miRNAs/isomiR and tRF Signatures of Colon Cancers

**Figure 4:**
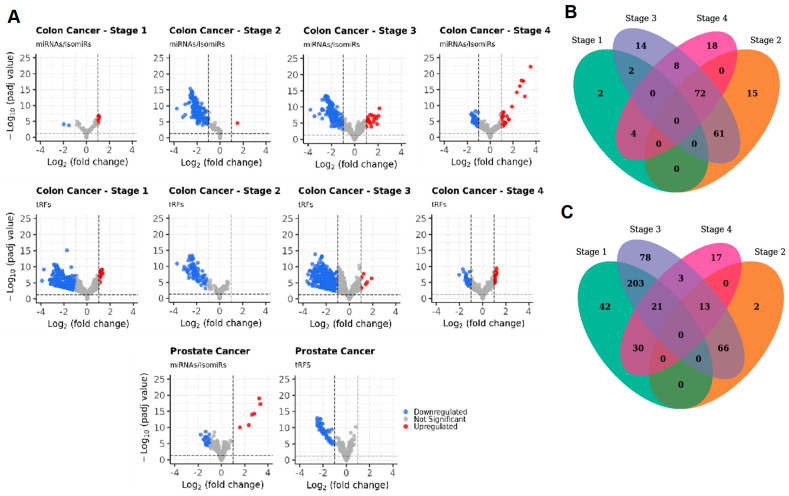
(**A**) Volcano plots of significantly differentially expressed miRNAs/isomiRs and tRFs. The number of common, significantly differentially expressed miRNAs (**B**) and tRFs (**C**) between different stages of colorectal cancer.

To determine the specific miRNA(s) associated with colorectal cancer, we compared 50 healthy control samples versus 100 patients with colorectal cancer (GSE71008) (Table 1). The colorectal cancer cohort comprised 25 samples from each stage of the disease: stage 1, stage 2, stage 3, and stage 4 (100 samples in total). Our analysis detected a total of 3537 miRNAs and isomiRs. Among the stages analyzed, the number of differentially expressed sequences was highest at stage 3, with 157 (Figure 2). The analysis revealed that the number of downregulated miRNAs and isomiRs exceeded the number of upregulated sequences in all stages except stage 1. Stage 2 exhibited the highest number of downregulated miRNAs and isomiRs, with only one sequence being upregulated. In contrast, stage 4 showed the largest number of upregulated sequences. This differential expression highlights the varying molecular profiles associated with each cancer stage, providing insights into the progression of colorectal cancer. No common miRNAs or isomiRs were detected across all stages of colorectal cancer. However, stages 2, 3, and 4 shared a total of 72 differentially expressed miRNAs and isomiRs (Figure 4B).

A total of 6957 tRFs were detected, with the highest number of differentially expressed tRFs 384 identified in stage 3 (Figure 1 and Figure 3B). Specifically, a greater number of tRFs were downregulated compared to upregulated sequences across all stages. Stage 3 exhibited the highest proportion of downregulated tRFs, with only 7 upregulated sequences. Conversely, stage 4 demonstrated the greatest number of upregulated sequences. Similar to miRNAs/isomiRs distribution, no common tRFs were detected across all stages; however, stages 1, 3, and 4 shared a total of 21 differentially expressed tRFs. Furthermore, stages 2, 3, and 4 shared another set of 13 common tRFs (Figure 4C). To visualize the differences observed and the significant changes in expression patterns, a volcano plot was generated to illustrate the differential expression of miRNAs/isomiRs, and tRFs across the various cancer stages (Figure 4A, upper eight plots). These findings highlight the distinct expression patterns of miRNAs, isomiRs, and tRFs at different stages of colorectal cancer. Interestingly, the two most significantly differentially expressed miRNAs with the highest abundance (stage 1 colorectal cancer) are the non-templated isomiRs: miR-129-5p_chr7(+)_chr11(+)_t_0_-3_nont_0_+3_GGC and miR-99b-5p_chr19(+)_t_0_-4_nont_0_+3_GGC. Among the miRNA families, the miR-99 family exhibited the lar-gest number of significant differentially expressed variants, including three non-templated isoforms. ROC curves identified miR-129-5p_chr7(+)_chr11(+)_t_0_3_nont_0_+3_GGC as the most accurate biomarker for distinguishing stage 1 colorectal cancer. This was followed by miR-99b-5p_chr19(+)_t_0_-4_nont_0_+3_GGC, miR-99a-5p_chr21(+)_t_0_-3_nont_0_+2_AG, miR-100-5p_chr11(-)_t_0_-4_nont_0_+3_GGT, and miR-143-3p_chr5(+)_t_0_-3_nont_0_+3_GTC among all significant differentially expressed miRNAs (Supplementary Figures S1–S3).

The top 10 most significantly differentially expressed tRFs annotated with Valine (Val), Glycine (Gly), and Glutamic Acid (Glu), as well as the top 3 most abundant diffe-rentially expressed tRFs annotated with Glycine (Gly), were identified. Among all signi-ficant differentially expressed tRFs, ROC curve analysis highlighted tDR-1:23-Val-AAC-1-M8-G12U as the most accurate biomarker for differentiating stage 1 colorectal cancer, achieving an AUC of 92% (Supplementary Figures S3–S5).

In stage 2 colorectal cancer, the top five most significantly differentially expressed miRNAs are non-templated isomiRs, three of which belong to the miR-30 family. Among them, let-7i-5p_chr12(+)_t_0-2_nont_0_+1_G and miR-99a-5p_chr21(+)_t_0_2_nont_0_+1_G are notable, with the latter being the isomiR with the highest abundance. The miRNA families let-7 and miR-181-5p contain the largest numbers of significantly differentially expressed variants, with 12 and 9 non-templated isoforms, respectively. Additionally, the miR-99-5p family includes eight non-templated and one templated isomiRs. ROC curve analysis identified miR-148b-3p_chr12(+)_t_0_-1_nont_0_+1_G as the most accurate differentiator of stage 2 colorectal cancer. It was followed by miR-30d-5p_nont_0_+2_CG, miR-30a-5p_nont_0_+2_CG, miR-25-3p_chr7(-)_t_0_-3_nont_0_+2_CG, and miR-143-3p_chr5(+)_t_0_2_nont_0_+1_G among all significantly differentially expressed miRNAs (Supplementary Figures S1–S3).

The top 10 most significantly differentially expressed tRFs were annotated with Hi-stidine (His), Glycine (Gly), and Glutamic Acid (Glu), while the top 3 most abundant differentially expressed tRFs were annotated with Glycine (Gly) and Glutamic Acid (Glu). Among all significant differentially expressed tRFs, tDR-1:26-Glu-TTC-2-U26G demonstrated the highest accuracy in differentiating stage 1 colon cancer, achieving an AUC of 79.8% based on ROC curve analysis (Supplementary Figure S3–S5).

In stage 3 colorectal cancer, the top five most significant differentially expressed miRNAs identified were all non-templated isomiRs: miR-26a-5p_chr12(-)_chr3(+)_t_0_-1_nont_0_+1_G and miR-30d-5p_nont_0_+2_CG, followed by miR-381-3p_chr14(+)_t_0_-1_nont_0_+1_G, miR-148a-3p_chr7(-)_t_0_-1_nont_0_+1_G, and miR-125a-5p_chr19(+)_t_0_-3_nont_0_+1_G. Notably, the last three isomiRs were also identified as the top performers in ROC analyses, achieving the highest AUC values among the top five isomiRs. The three most abundant differentially expressed miRNAs were miR-99a-5p_chr21(+)_t_0_-2_nont_0_+1_G, miR-22-3p_chr17(-)_t_0_-1_nont_0_+1_G, and miR-181a-5p_chr1(-)_chr9(+)_t_0_-3_nont_0_+1_G. Among the miRNAs with the largest number of si-gnificantly differentially expressed variants, let-7, miR-181-5p, miR-99-5p, and miR-1246 stood out with 12, 9, 9, and 8 isomiRs, respectively. Notably, all variants within these miRNA families were non-templated, except for one templated variant each for miR-99-5p and miR-1246, as well as the canonical form of miR-1246. ROC curve analyses revealed that miR-22-3p_nont_0_+3_AGA was the most accurate discriminator of stage 3 colorectal cancer, followed by miR-125a-5p_chr19(+)_t_0_-3_nont_0_+1_G, miR-381-3p_chr14(+)_t_0_-1_nont_0_+1_G, miR-148a-3p_chr7(-)_t_0_-1_nont_0_+1_G, and miR-218-5p_chr5(-)_chr4(+)_t_0_-1_nont_0_+1_G among all significantly differentially expressed miRNAs (Supplementary Figures S1–S3).

The top 10 most significant differentially expressed tRFs were annotated with Glycine (Gly), Glutamic Acid (Glu), and Valine (Val), while the top 3 most abundant diffe-rentially expressed tRFs were annotated with Glycine (Gly) and Glutamic Acid (Glu). ROC curves identified tDR-1:33-Glu-CTC-1-M2-U20G as the most accurate differentiator of stage 1 from colorectal cancer with 84.3% AUC among all significant differentially expressed tRFs (Supplementary Figures S3–S5).

In stage 4 colorectal cancer, the top seven most significantly differentially expressed miRNAs all originated from miR-1246, including both non-templated isoforms and the canonical form. The three most abundant significant differentially expressed miRNAs were miR-99a-5p_chr21(+)_t_0_-2_nont_0_+1_G, miR-486-5p_chr8(-)_chr8(+)_t_0_-2, and miR-22-3p_chr17(-)_t_0_-1_nont_0_+1_G. Among miRNA families, those with the most significant differentially expressed variants were let-7, miR-99-5p, miR-181-5p, and miR-1246, with 9, 8, 8, and 8 variants, respectively. All variants were non-templated isoforms except for one templated isoform and the canonical form of miR-1246. ROC curve analysis identified miR-1246_nont_0_+2_GA as the most accurate discriminator of stage 4 colore-ctal cancer, followed by miR-99a-5p_chr21(+)_t_0_-1_nont_0_+3_AGA, miR-1246_nont_0_+1_G, miR-100-5p_chr11(-)_t_0_-4_nont_0_+3_AGT, and miR-1246_chr2(-) among all significant differentially expressed miRNAs (Supplementary Figures S1–S3).

The top 10 most significantly differentially expressed tRFs annotated with Glycine (Gly) and Glutamic Acid (Glu), along with the top 3 most abundant differentially expressed tRFs in the same categories, were identified. Among these, ROC curve analysis highlighted tDR-1:33-Glu-CTC-1-M2-U13A as the most accurate biomarker for distinguishing stage 1 colorectal cancer, achieving an AUC of 81.4% (Supplementary Figures S3–S5).

### 2.2. Discovery of the miRNA/isomiRand tRF Signatures Related to Prostate Cancer

To identify the specific miRNAs and tRNAs associated with prostate cancer, we compared 50 healthy control samples with 36 prostate cancer samples from the GSE71008 dataset. The prostate cancer samples were further classified into two groups: 15 samples from patients with hormone-sensitive prostate cancer (HSPC) and 21 samples from patients with castration-resistant prostate cancer (CRPC). A total of 2.965 differentially expressed miRNAs were identified, including two canonical miRNAs and 32 isomiRs. Of these, 6 miRNAs were upregulated and 28 were downregulated in the prostate cancer group. A volcano plot (Figure 4A, lower left plot) was generated to visualize the differe-ntial expression of miRNAs. Additionally, 5.322 differentially expressed tRNA-derived fragments (tRFs) were detected, of which only 55 were significantly differentially expressed, and all of these were downregulated (Figure 4A, lower right plot).

The five most significantly differentially expressed individual isomiRs were miR-1246_nont_0_+3_GAG, miR-1246_nont_0_+1_G, miR-1246_chr2(-)_t_+1_0_nont_0_+2_GA, miR-1246_chr2(-)_t_+1_0_nont_0_+3_GAG, and miR-1246_chr2(-)_t_+1_0_nont_0_+1_G. The top five most abundant, significant differentially expressed isomiRs were miR-433-3p_chr14(+)_t_0_-1_nont_0_+1_G, miR-128-3p_chr2(+)_chr3(+)_t_0_-1_nont_0_+1_G, miR-92a-3p_chr13(+)_chrX(-)_t_0_-1_nont_0_+1_G, miR-6529-5p_chr3(-)_t_0_-2_nont_0_+1_G, and miR-128-3p_chr2(+)_chr3(+)_t_0_-2_nont_0_+1_G. The miRNA with the highest number of significant differentially expressed variants was miR-1246, which had five non-templated isoforms. ROC curves identified miR-451a_chr17(-)_t_0_+3 and miR-144-5p_chr17(-) as the most accurate discriminators of prostate cancer, with diagnostic accuracy of approximately 80%. These were followed by miR-584-5p_chr5(-)_t_0_-2_nont_0_+1_T, miR-144-5p_chr17(-)_t_0_+1, and miR-584-5p_chr5(-)_t_0_-1, among all significant differentially expressed isomiRs (Supplementary Figures S1–S3).

The top 10 most significantly differentially expressed tRFs, annotated with Glycine (Gly) and Glutamic Acid (Glu), and the top 3 most abundant differentially expressed tRFs, also annotated with these amino acids, were identified. ROC curve analysis revealed that tDR-1:35-Val-AAC-1-M6-A34G exhibited the highest accuracy as a differentiator, with an AUC of 83.7% among all significant differentially expressed tRFs (Supplementary Figures S3–S5).

### 2.3. Biomarkers Identified Through Combinatorial Analysis

Combining the prognostic and diagnostic capabilities of two or more potential bio- markers enhances the statistical robustness and yields improved results, not only in di-stinguishing healthy individuals from cancer patients but also in differentiating between various stages of the same cancer type. The combination of various molecules, including isomiRs and tRFs, as biomarkers for colorectal cancer demonstrated robust results, parti-cularly in stages 1 and 4. In stage 1, the most statistically significant combination consisted solely of tRFs annotated with tRNAs of Glycine (Gly) and Glutamic acid (Glu), achieving minimum accuracy, AUC, sensitivity, and specificity of 82%, 72%, 50%, and 83%, respe-ctively (Table 2). The top combinations predominantly featured tRFs corresponding to tRNAs-Val, -Gly, -Glu, and -His variants. In stage 2, the best combination included both, isomiRs and tRFs (miR-30e-5p, let-7g-5p, and Gly), with minimum values for accuracy, AUC, sensitivity, and specificity of 60%, 60%, 50%, and 56%, respectively. The top combinations featured isomiRs and tRFs, with a dominance of miR-30d-5p, miR-30a-5p, tRNA-Glu, and tRNA-Gly variants. For stage 3, the optimal combination comprised both isomiRs and tRFs (miR-128-3p, Gly, and Val), with minimum accuracy, AUC, sensitivity, and specificity of 78%, 83%, 100%, and 64%, respectively. The top combinations included isomiRs and tRFs, with a dominance of miR-22-3p, miR-9-5p, and tRNA-Glu variants. In stage 4, the best combination of isomiRs and tRFs (miR-125b-5p, miR-1246, and Gly) showed minimum accuracy, AUC, sensitivity, and specificity of 87%, 75%, 50%, and 100%, respectively. The top combinations featured isomiRs and tRFs, with a dominance of miR-99a-5p variants (Table 2).

In prostate cancer, the best combination included isomiRs and tRFs (of miR-584-5p, Glu, and Val), yielding minimum accuracy, AUC, sensitivity, and specificity of 80%, 80%, 80%, and 64%, respectively. The top combinations were dominated by isomiRs and tRFs, with miR-584-5p, miR-144-5p, Glu, and Val variations dominating (Table 2).

## 3. Discussion

In our study, we utilized CombiROC to identify a comprehensive panel of bio-markers, including miRNAs, isomiRs, and tRFs, to achieve optimal diagnostic accuracy for colorectal and prostate cancers. Previous research has successfully employed CombiROC to determine the effective combinations of miRNA markers from fluid samples for various cancers, such as primary central nervous system lymphoma [31], lung adenocarcinoma [32], and lung squamous cell carcinoma [33]. These studies have established that circulating miRNAs can be reliable biomarkers for differentiating cancer patients from healthy individuals. In our investigation, we expanded the scope of potential biomarkers beyond miRNAs by incorporating the analysis of isomiRs and tRFs. Relying on the capabilities of CombiROC, we computed all possible combinations of these small RNA mar-kers to identify the most effective biomarker panels that enhance diagnostic accuracy. We further assessed the performance of these marker combinations through ROC curve ana-lysis, which enabled us to distinguish effectively between cancer patients and healthy controls, achieving an accuracy rate of approximately 80%.

The role of specific miRNAs in colorectal cancer has been well-documented in tissue biopsies, with some miRNAs acting as oncogenes or tumor suppressors, depending on their expression context. Some of them can correlate with advanced stages of the disease, while other miRNAs may be downregulated due to epigenetic modifications [34,35,36]. Here, we report the dynamics of miRNAs and their sequence variants in liquid biopsy. Expression analysis of miRNAs and their variants in EVs at successive stages of colorectal cancer indicates that the accumulation of non-templated isoforms is significantly affected. Notably, changes in the number and sequence of these isoforms are more pronounced in stages 2, 3, and 4 compared to stage 1. A striking observation is the substantial depletion of non-templated isoforms that terminate with guanine (G) at the 3′ end. This trend is particularly evident at stage 2, where most differentially expressed isoforms are downregulated, except for one, and nearly all end with guanine. It remains unclear whether this phenomenon results from cancer-related alterations in the biogenesis of these isoforms or their selective packaging in EVs.

A significant observation from this study is the similarity among stages 2, 3, and 4 of colorectal cancer, characterized by 72 common differentially expressed isomiRs. Notable variants include those of miR-128-3p, miR-125-5p, miR-99a-5p, let-7a-5p, let-7b-5p, miR-30a-5p, miR-30d-5p, miR-30e-5p, and miR-22-3p. This study confirms the differential expression of miR-128-5p and miR-125-5p, aligning with the findings of Yuan et al. (2016) [37]. Importantly, it reveals that only specific isomiRs contribute to their expression, while canonical forms are excluded. Several of these miRNAs have been linked to tumor progression and metastasis in colorectal cancer. For instance, miR-99a-5p, let-7a-5p, and let-7b-5p are associated with advanced disease stages and distant metastasis [38]. Additio-nally, miR-22-3p has been shown to suppress cell proliferation, migration, and invasion in colorectal cancer [39]. Another important finding is the presence of miR-1246 in stages 3 and 4, represented by various isomiRs. Recent studies involving liquid biopsies have identified miR-1246 as a potential biomarker for colorectal cancer [40]. This miRNA is re-cognized for its oncogenic role across multiple cancer types, including colorectal, breast, and pancreatic cancers [41]. It has been validated through PCR techniques and has shown promise in distinguishing between chemosensitive and chemoresistant patients with co-lorectal cancer [42,43]. Furthermore, both miR-30a-5p and miR-99a-5p are characterized as exosomal miRNAs that play crucial roles in regulating key genes involved in tumor growth and suppression [44]. Their influence on tumor dynamics emphasizes the potential of these miRNAs as therapeutic targets and biomarkers in colorectal cancer ma-nagement.

Regarding tRFs detected in analyzed datasets, variants of tRNA-Glu are among the top candidates for potential biomarkers and are significantly differentially expressed across all stages. The literature suggests that the overexpression of tRNA-Glu-GCC in the blood can contribute to the increased proliferation of colorectal cancer cells [45].

In line with previous studies on exosomal biomarkers in prostate carcinoma, we ide-ntified several differentially expressed miRNAs, notably the upregulated miR-125a-5p and miR-219a-5p, each associated with a specific non-templated isomiR [37]. Another study highlighted the role of miR-1246, revealing that its overexpression in a prostate cancer cell line inhibited tumor growth, proliferation, invasiveness, and migration [46]. Furthermore, the overexpression of miR-451a was shown to significantly suppress prostate cell proliferation, migration, and invasion [47], while decreased expression of miR-451a was associated with metastasis, as it was observed in metastatic prostate cancer tissues and cell lines [48]. Additionally, the overexpression of miR-144 induced tumor cell death, with several studies confirming its role in high-grade tumors through involvement in va-rious cellular pathways [49,50,51].

Recent studies have validated the existence and significance of tRFs in prostate cancer, highlighting their potential utility in both tissue samples and liquid biopsies [52]. Notably, specific tRFs derived from tRNA-Glu-TTC and tRNA-Val-CAC have garnered attention for their roles in this context [53,54].

## 4. Materials and Methods

In our study, we focused on two different types of cancer, colorectal and prostate cancer. To analyze the molecular profiles of the EVs associated with these cancers, we collected samples from the EVAtltas database [30]. We used the raw sequencing reads and data published under the GEO repository for the above cancer types with ID accession: GSE71008 (Table 1) [37].

### 4.1. Identification and Classification of miRNAs/isomiRs & tRFs

Recently, we contributed to the miRNA field by developing miRGalaxy, an open-source, Galaxy-based framework for in-depth miR and isomiR analysis from NGS data [55]. We will use the miRGalaxy pipeline for our study by performing minor changes on the pipeline to accommodate the detection and identification of tRFs. The first steps of a quality check (QC) and adapter trimming remain the same, and the default options were used in the ArmDB tool—miRBase (v22) as a miR source database, *Homo sapiens* hg38 genome, and 6nt extensions of the RefSeq miR sequences [56]. Then, alignment was performed with Bowtie by using the gtRNADB (v21) database as a reference for the detection of tRFs, and the unmapped reads re-aligned with Bowtie by using the custom ArmDB database as a reference for the detection of miRNAs [57,58]. The quantification of tRFs was performed in R (4.1.2) and miRNAs by using the isoRead tool according to the miRGalaxy pipeline. The tDRnammer tool (1.3.1) provided consistent and stable names for annotating tRFs. In addition, tRFs were classified into groups (5′-tRFs, 3′-tRFs) based on the classification system established by Kumar et al. [18]. This system was further enriched with two additional groups: ‘internal’ tRFs (i-tRFs), defined as fragments with cleavage from 30 to 52 nucleotides, and tRFs, which include part of the V-loop, following Sprinzl et al.’s tRNA position numbering system as implemented in the tDRnammer tool [59,60]. Finally, tRFs and miRNAs with a low number of counts were filtered in R by using the function filterbyexp, black and white cases were also retained, allowing 0 values in a maximum of 50% of the samples group, and differential expression was performed by using Deseq2 in R [61]. Differentially expressed miRNAs and tRNAs were analyzed together using a cutoff of p adjusted value (padj) < 0.05 and 1 < |Log2FC| < 10. Biomarker candidates of miRNAs and tRFs were then tested using Cox regression analysis and receiver operating chara-cteristics (ROC) curves individually and combined using the ROCR package “https://cran.r-project.org/web/packages/ROCR/ROCR.pdf” (accessed on 7 February 2025) in R/Bioconductor.

### 4.2. Combinatorial Analysis and Evaluation of Biomarkers

The combination of potential biomarkers consists of miRNAs and tRFs performed by using CombiROC package in R (4.1.2), which determines the optimal combinations of the biomarkers from complex omics data. During the combinatorial analysis, the maximum number of combinations was set to three. The ‘signalthr’ parameter of the ‘combi’ function was determined using density plots, while ‘combithr’, set to three, was the most stringent value for achieving more precise results. All optimal combinations with sensitivity or specificity below 20 were discarded using the CombiROC package [31]. In addition, due to CombiROC using a GLM model approach, 500 runs of the model were performed by splitting the input data into training (70% of data) and test sets (30% of data) for validation, using the Monte Carlo Simulation method to improve the reliability of the results. The evaluation of the statistical model was conducted by calculating metrics such as accuracy, AUC, sensitivity, and specificity using the confusionMatrix function from the caret pa-ckage in R. As input datasets for the combinatorial analysis, the counts of the top 20 si-gnificantly differentially expressed miRNAs and tRFs with the highest AUC values were used.

## 5. Conclusions

In conclusion, this study provides a comprehensive combinatorial analysis of isomiRs and tRFs as potential biomarkers for detecting two cancer types and the four cancer stages through liquid biopsies. By using advanced bioinformatics tools, we emphasize the importance of considering the synergistic effects of different RNA species. Unlike conve-ntional biomarkers, isomiRs and tRFs offer highly refined indicators of disease status, ca-pturing the dynamic and heterogeneous nature of cancer [7]. Their detectability in bio-fluids such as blood or urine enables the real-time monitoring of molecular changes, empowering clinicians to adapt therapeutic strategies to the evolving molecular landscape of tumors.

The joint analysis of isomiRs and tRFs has proven more effective in identifying robust and informative biomarkers than analyzing each RNA type in isolation. This combinatorial approach holds great potential to enhance the accuracy and sensitivity of cancer diagnosis and treatment.

However, further research is imperative to unravel the biological mechanisms dri-ving the dysregulation of isomiRs and tRFs in cancer. Additionally, the inconsistent nomenclature of tRFs across studies has confused and hindered cross-study comparisons [17,62]. A standardized and universally accepted nomenclature for tRFs is urgently needed. Finally, large-scale clinical studies are critical to validate the practical utility of these biomarkers in real-world settings.

## Figures and Tables

**Table 1:** Information about the sample data used in the analysis.

Samples	Groups	Number of Samples		Gender		Age	
		Per Group	Total	Male	Female	Average	Range
Colorectal Cancer	Stage 1	25	100	13	12	59	19–88
	Stage 2	25		12	13	39	15–71
	Stage 3	25		13	12	64	27–90
	Stage 4	25		13	12	58	26–82
Prostate Cancer	Hormone sensitive prostate cancer (HSPC)	15	36	36	NA	69	49–87
	Castration resistant prostate cancer (CRPC)	21					
Healthy Donors	Controls	50	50	25	25	54	25–79

**Table 2:** Best biomarkers combination per condition.

Cancer Type		Best Combinations	Minimum Values			
			Accuracy	AUC	Sensitivity	Specificity
Colorectal Cancer	Stage 1	tDR-1:33-Gly-GCC-1-C31G tDR-1:34-Glu-CTC-1-M2-U16A tDR-1:29-Gly-CCC-1-M4-C13G	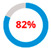	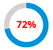	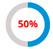	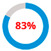
	Stage 2	tDR-1:32-Gly-GCC-1-C31G hsa-miR-30e-5p_nont_0_+2_CG hsa-let-7g-5p_chr3(-)_t_0_2_nont_0_+1 _G	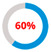	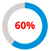	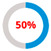	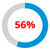
	Stage 3	tDR-1:34-Gly-GCC-2-M3-U16A tDR-1:23-Val-AAC-1-M8-G12U hsa-miR-128-3p_chr2(+)_chr3(+)_t_0_-1_nont_0_+1_G	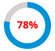	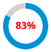	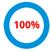	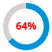
	Stage 4	tDR-1:33-Gly-GCC-1-C31G hsa-miR-1246_nont_0_+2_GA hsa-miR-125b-5p_chr11(-)_chr21(+)_t_0_-1_nont_0_+1_G	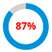	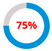	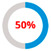	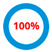
Prostate Cancer		tDR-1:31-Glu-CTC-1-M2-A24C tDR-1:35-Val-AAC-1-M6-A34G hsa-miR-584-5p_chr5(-)_t_0_-2_nont_0_+1_T	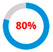	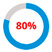	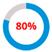	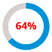

## Data Availability

Data are contained within the article and Supplementary Materials.

## Supplementary Materials

The following supporting information can be downloaded at: https://www.mdpi.com/article/10.3390/ncrna11010017/s1, Figure S1: Top 5 differentially expressed miRNAs with the highest AUC per condition; Figure S2: Normalized counts distribution of top 5 differentially expressed miRNAs with the highest AUC per condition; Figure S3: Most abundant significant differentially expressed miRNAs/isomiRs and tRFs per condition; Figure S4: Top 5 differentially expressed tRFs with the highest AUC per condition; Figure S5: Normalized counts distribution of top 5 differentially expressed tRFs with the highest AUC per condition; Table S1: Results of combinatorial analysis; Table S2: Results of significantly differentially expressed miRNAs/isomiRs and tRFs.

## References

[1] Lone S.N., Nisar S., Masoodi T., Singh M., Rizwan A., Hashem S., El-Rifai W., Bedognetti D., Batra S.K., Haris M. (2022). Liquid biopsy: A step closer to transform diagnosis, prognosis and future of cancer treatments. Mol. Cancer.

[2] Glogovitis I., Yahubyan G., Würdinger T., Koppers-Lalic D., Baev V. (2021). Isomirs–hidden soldiers in the mirna regulatory army, and how to find them?. Biomolecules.

[3] Wilusz J.E. (2015). Controlling translation via modulation of tRNA levels. Wiley Interdiscip. Rev. RNA.

[4] Santos M., Fidalgo A., Varanda A.S., Oliveira C., Santos M.A.S. (2019). tRNA Deregulation and Its Consequences in Cancer. Trends Mol. Med..

[5] Distefano R., Tomasello L., Vinciguerra G.L.R., Gasparini P., Xiang Y., Bagnoli M., Marceca G.P., Fadda P., Laganà A., Acunzo M. (2022). Pan-Cancer Analysis of Canonical and Modified miRNAs Enhances the Resolution of the Functional miRNAome in Cancer. Cancer Res..

[6] Drury R.E., O’Connor D., Pollard A.J. (2017). The clinical application of MicroRNAs in infectious disease. Front. Immunol..

[7] Zelli V., Compagnoni C., Capelli R., Corrente A., Cornice J., Vecchiotti D., Di Padova M., Zazzeroni F., Alesse E., Tessitore A. (2021). Emerging role of isomiRs in cancer: State of the art and recent advances. Genes.

[8] Liu H., Lei C., He Q., Pan Z., Xiao D., Tao Y. (2018). Nuclear functions of mammalian MicroRNAs in gene regulation, immunity and cancer. Mol. Cancer.

[9] Telonis A.G., Loher P., Jing Y., Londin E., Rigoutsos I. (2015). Beyond the one-locus-one-miRNA paradigm: microRNA isoforms enable deeper insights into breast cancer heterogeneity. Nucleic Acids Res..

[10] Tomasello L., Distefano R., Nigita G., Croce C.M. (2021). The MicroRNA Family Gets Wider: The IsomiRs Classification and Role. Front. Cell Dev. Biol..

[11] Agnelli L., Bisognin A., Todoerti K., Manzoni M., Taiana E., Galletti S., Cutrona G., Gaffo E., Bortoluzzi S., Neri A. (2019). Expanding the repertoire of miRNAs and miRNA-offset RNAs expressed in multiple myeloma by small RNA deep sequencing. Blood Cancer J..

[12] Saikia M., Hatzoglou M. (2015). The many virtues of tRNA-derived stress-induced RNAs (tiRNAs): Discovering novel mechanisms of stress response and effect on human health. J. Biol. Chem..

[13] Deng L., Wang H., Fan T., Chen L., Shi Z., Mi J.L., Huang W.M., Wang R., Hu K. (2022). Potential Functions of the tRNA-Derived Fragment tRF-Gly-GCC Associated With Oxidative Stress in Radiation-Induced Lung Injury. Dose-Response.

[14] Di Fazio A., Schlackow M., Pong S.K., Alagia A., Gullerova M. (2022). Dicer dependent tRNA derived small RNAs promote nascent RNA silencing. Nucleic Acids Res..

[15] Fagan S.G., Helm M., Prehn J.H.M. (2021). tRNA-derived fragments: A new class of non-coding RNA with key roles in nervous system function and dysfunction. Prog. Neurobiol..

[16] Tao E.W., Wang H.L., Cheng W.Y., Liu Q.Q., Chen Y.X., Gao Q.Y. (2021). A specific tRNA half, 5’tiRNA-His-GTG, responds to hypoxia via the HIF1α/ANG axis and promotes colorectal cancer progression by regulating LATS2. J. Exp. Clin. Cancer Res..

[17] Lee Y.S., Shibata Y., Malhotra A., Dutta A. (2009). A novel class of small RNAs: tRNA-derived RNA fragments (tRFs). Genes Dev..

[18] Kumar P., Anaya J., Mudunuri S.B., Dutta A. (2014). Meta-analysis of tRNA derived RNA fragments reveals that they are evolutionarily conserved and associate with AGO proteins to recognize specific RNA targets. BMC Biol..

[19] Kumar P., Kuscu C., Dutta A. (2016). Biogenesis and Function of Transfer RNA-Related Fragments (tRFs). Trends Biochem. Sci..

[20] Li S., Xu Z., Sheng J. (2018). tRNA-derived small RNA: A novel regulatory small non-coding RNA. Genes.

[21] Shen Y., Yu X., Zhu L., Li T., Yan Z., Guo J. (2018). Transfer RNA-derived fragments and tRNA halves: Biogenesis, biological functions and their roles in diseases. J. Mol. Med..

[22] Zhu L., Ge J., Li T., Shen Y., Guo J. (2019). tRNA-derived fragments and tRNA halves: The new players in cancers. Cancer Lett..

[23] Fu M., Gu J., Wang M., Zhang J., Chen Y., Jiang P., Zhu T., Zhang X. (2023). Emerging roles of tRNA-derived fragments in cancer. Mol. Cancer.

[24] Weng Q., Wang Y., Xie Y., Yu X., Zhang S., Ge J., Li Z., Ye G., Guo J. (2022). Extracellular vesicles-associated tRNA-derived fragments (tRFs): Biogenesis, biological functions, and their role as potential biomarkers in human diseases. J. Mol. Med..

[25] Tosar J.P., Cayota A. (2020). Extracellular tRNAs and tRNA-derived fragments. RNA Biol..

[26] Calin G.A., Dumitru C.D., Shimizu M., Bichi R., Zupo S., Noch E., Aldler H., Rattan S., Keating M., Rai K. (2002). Frequent deletions and down-regulation of micro-RNA genes miR15 and miR16 at 13q14 in chronic lymphocytic leukemia. Proc. Natl. Acad. Sci. USA.

[27] Weber J.A., Baxter D.H., Zhang S., Huang D.Y., Huang K.H., Lee M.J., Galas D.J., Wang K. (2010). The microRNA spectrum in 12 body fluids. Clin. Chem..

[28] Rubio M., Bustamante M., Hernandez-Ferrer C., Fernandez-Orth D., Pantano L., Sarria Y., Piqué-Borras M., Vellve K., Agramunt S., Carreras R. (2018). Circulating miRNAs, isomiRs and small RNA clusters in human plasma and breast milk. PLoS ONE.

[29] Zhang Y., Deng Q., Tu L., Lv D., Liu D. (2020). TRNA-derived small RNAs: A novel class of small RNAs in human hypertrophic scar fibroblasts. Int. J. Mol. Med..

[30] Liu C.J., Xie G.Y., Miao Y.R., Xia M., Wang Y., Lei Q., Zhang Q., Guo A.Y. (2022). EVAtlas: A comprehensive database for ncRNA expression in human extracellular vesicles. Nucleic Acids Res..

[31] Mazzara S., Rossi R.L., Grifantini R., Donizetti S., Abrignani S., Bombaci M. (2017). CombiROC: An interactive web tool for selecting accurate marker combinations of omics data. Sci. Rep..

[32] Abdipourbozorgbaghi M., Vancura A., Radpour R., Haefliger S. (2024). Circulating miRNA panels as a novel non-invasive diagnostic, prognostic, and potential predictive biomarkers in non-small cell lung cancer (NSCLC). Br. J. Cancer.

[33] Bica C., Jurj A., Harangus A., Ciocan C., Moldovan A., Zanoaga O., Burz C., Ferracin M., Raduly L., Berindan-Neagoe I. (2024). miRNA patterns in male LUSC patients—The 3-way mirror: Tissue, plasma and exosomes. Transl. Oncol..

[34] Schetter A.J., Okayama H., Harris C.C. (2012). The role of microRNAs in colorectal cancer. Cancer J..

[35] Tâlvan C.D., Tâlvan E.T., Mohor C.I., Budișan L., Grecu V., Mihalache M., Zănoagă O., Chira S., Berindan-Neagoe I., Cristea V. (2024). Exploring miRNA Profiles in Colon Cancer: A Focus on miR101-3p, miR106a-5p, and miR326. Cancers.

[36] Zhang N., Hu X., Du Y., Du J. (2021). The role of miRNAs in colorectal cancer progression and chemoradiotherapy. Biomed. Pharmacother..

[37] Yuan T., Huang X., Woodcock M., Du M., Dittmar R., Wang Y., Tsai S., Kohli M., Boardman L., Patel T. (2016). Plasma extracellular RNA profiles in healthy and cancer patients. Sci. Rep..

[38] Chen Y., Liu H., Ning S., Wei C., Li J., Wei W., Zhang L. (2022). The High Ratio of the Plasma miR-96/miR-99b Correlated with Poor Prognosis in Patients with Metastatic Colorectal Cancer. Front. Mol. Biosci..

[39] Cong J., Gong J., Yang C., Xia Z., Zhang H. (2020). miR-22 suppresses tumor invasion and metastasis in colorectal cancer by targeting NLRP3. Cancer Manag. Res..

[40] Salah M., Shaheen I.A., El-Shanawany P., Saad N.E., Saad R., El Guibaly M., Momen N. (2020). Detection of miR-1246, miR-23a and miR-451 in sera of colorectal carcinoma patients: A case-control study in Cairo University Hospital. Afr. Health Sci..

[41] Ghafouri-Fard S., Hussen B.M., Badrlou E., Abak A., Taheri M. (2021). MicroRNAs as important contributors in the pathogenesis of colorectal cancer. Biomed. Pharmacother..

[42] Dos Santos K.A., Dos Santos I.C.C., Silva C.S., Ribeiro H.G., De Farias Domingos I., Silbiger V.N. (2021). Circulating exosomal mirnas as biomarkers for the diagnosis and prognosis of colorectal cancer. Int. J. Mol. Sci..

[43] Ogata-Kawata H., Izumiya M., Kurioka D., Honma Y., Yamada Y., Furuta K., Gunji T., Ohta H., Okamoto H., Sonoda H. (2014). Circulating exosomal microRNAs as biomarkers of colon cancer. PLoS ONE.

[44] Bakhsh T., Alhazmi S., Farsi A., Yusuf A.S., Alharthi A., Qahl S.H., Alghamdi M.A., Alzahrani F.A., Elgaddar O.H., Ibrahim M.A. (2024). Molecular detection of exosomal miRNAs of blood serum for prognosis of colorectal cancer. Sci. Rep..

[45] Wu J., Hu S., Zhang L., Xin J., Sun C., Wang L., Ding K., Wang B. (2020). Tumor circulome in the liquid biopsies for cancer diagnosis and prognosis. Theranostics.

[46] Bhagirath D., Yang T.L., Bucay N., Sekhon K., Majid S., Shahryari V., Dahiya R., Tanaka Y., Saini S., Affairs V. (2018). microRNA-1246 Is an Exosomal Biomarker for Aggressive Prostate Cancer. Cancer Res..

[47] Liu Y., Yang H.Z., Jiang Y.J., Xu L.Q. (2020). miR-451a is downregulated and targets PSMB8 in prostate cancer. Kaohsiung J. Med. Sci..

[48] Wang G., Yao L., Yang T., Guo L., Gu S., Liu J., Yang K. (2019). MiR-451 suppresses the growth, migration, and invasion of prostate cancer cells by targeting macrophage migration inhibitory factor. Transl. Cancer Res..

[49] Huang Y., Zhang H., Gu X., Qin S., Zheng M., Shi X., Peng C., Ju S. (2021). Elucidating the Role of Serum tRF-31-U5YKFN8DYDZDD as a Novel Diagnostic Biomarker in Gastric Cancer (GC). Front. Oncol..

[50] Zhang Y., Gu X., Qin X., Huang Y., Ju S. (2022). Evaluation of serum tRF-23-Q99P9P9NDD as a potential biomarker for the clinical diagnosis of gastric cancer. Mol. Med..

[51] Cui H., Li H., Wu H., Du F., Xie X., Zeng S., Zhang Z., Dong K., Shang L., Jing C. (2022). A novel 3’tRNA-derived fragment tRF-Val promotes proliferation and inhibits apoptosis by targeting EEF1A1 in gastric cancer. Cell Death Dis..

[52] Magee R.G., Telonis A.G., Loher P., Londin E., Rigoutsos I. (2018). Profiles of miRNA Isoforms and tRNA Fragments in Prostate Cancer. Sci. Rep..

[53] Liu Y., Chen W., Liu R., Lan D., Zhao J. Serum Trna-Derived Fragments (Trfs) as Biomarkers for Diagnosing Prostate Cancer. https://ssrn.com/abstract=4872443.

[54] Ferre-Giraldo A., Castells M., Sánchez-Herrero J.F., López-Rodrigo O., de Rocco-Ponce M., Bassas L., Vigués F., Sumoy L., Larriba S. (2024). Semen sEV tRF-Based Models Increase Non-Invasive Prediction Accuracy of Clinically Significant Prostate Cancer among Patients with Moderately Altered PSA Levels. Int. J. Mol. Sci..

[55] Glogovitis I., Yahubyan G., Würdinger T., Koppers-lalic D., Baev V. (2021). Mirgalaxy: Galaxy-based framework for interactive analysis of microrna and isomir sequencing data. Cancers.

[56] Griffiths-Jones S., Grocock R.J., van Dongen S., Bateman A., Enright A.J. (2006). miRBase: microRNA sequences, targets and gene nomenclature. Nucleic Acids Res..

[57] Langmead B., Trapnell C., Pop M., Salzberg S.L. (2009). Ultrafast and memory-efficient alignment of short DNA sequences to the human genome. Genome Biol..

[58] Chan P.P., Lowe T.M. (2009). GtRNAdb: A database of transfer RNA genes detected in genomic sequence. Nucleic Acids Res..

[59] Gauss D.H., Sprinzl M. (1983). Compilation of sequences of tRNA genes. Nucleic Acids Res..

[60] Holmes A.D., Chan P.P., Chen Q., Ivanov P., Drouard L., Polacek N., Kay M.A., Lowe T.M. (2023). A standardized ontology for naming tRNA-derived RNAs based on molecular origin. Nat. Methods.

[61] Love M.I., Huber W., Anders S. (2014). Moderated estimation of fold change and dispersion for RNA-seq data with DESeq2. Genome Biol..

[62] Balatti V., Nigita G., Veneziano D., Drusco A., Stein G.S., Messier T.L., Farina N.H., Lian J.B., Tomasello L., Liu C.-G. (2017). tsRNA signatures in cancer. Proc. Natl. Acad. Sci. USA.

