# Supplementary material for: Combinatorial Analysis of miRNAs and tRNA Fragments as Potential Biomarkers for Cancer Patients in Liquid Biopsies
Source: Noncoding RNA. 2025 Feb 14;11(1):17. doi: 10.3390/ncrna11010017 (PMC11858735; doi:10.3390/ncrna11010017)
Supplement: Supplementary file 1 [file ncrna-11-00017-s001.zip › Figure S2.pdf]

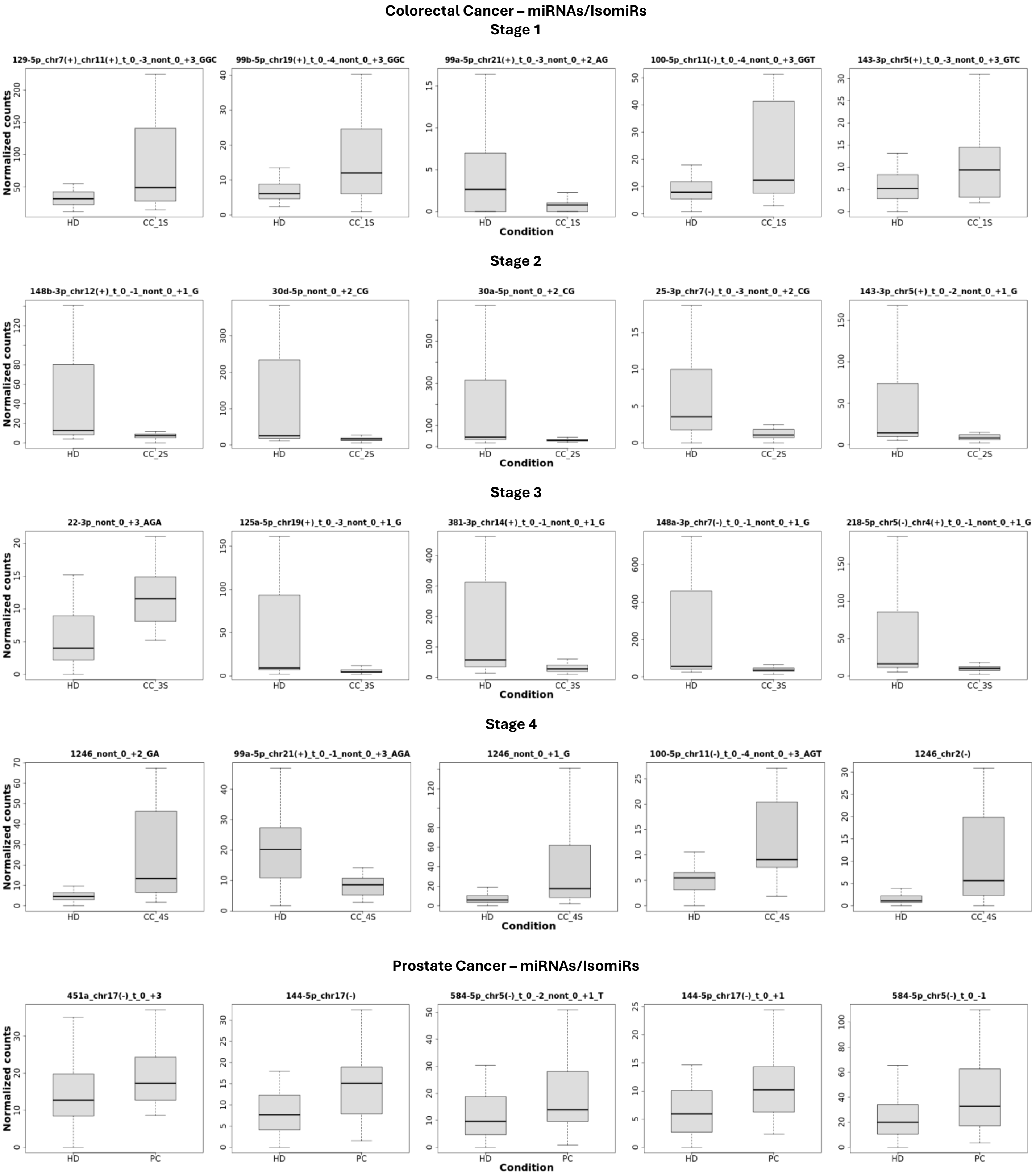

Figure S2. Normalized counts distribution of top 5 differentially expressed miRNAs/IsomiRs with the highest AUC per condition.
