# Supplementary material for: Combinatorial Analysis of miRNAs and tRNA Fragments as Potential Biomarkers for Cancer Patients in Liquid Biopsies
Source: Noncoding RNA. 2025 Feb 14;11(1):17. doi: 10.3390/ncrna11010017 (PMC11858735; doi:10.3390/ncrna11010017)
Supplement: Supplementary file 1 [file ncrna-11-00017-s001.zip › Figure S3.pdf]

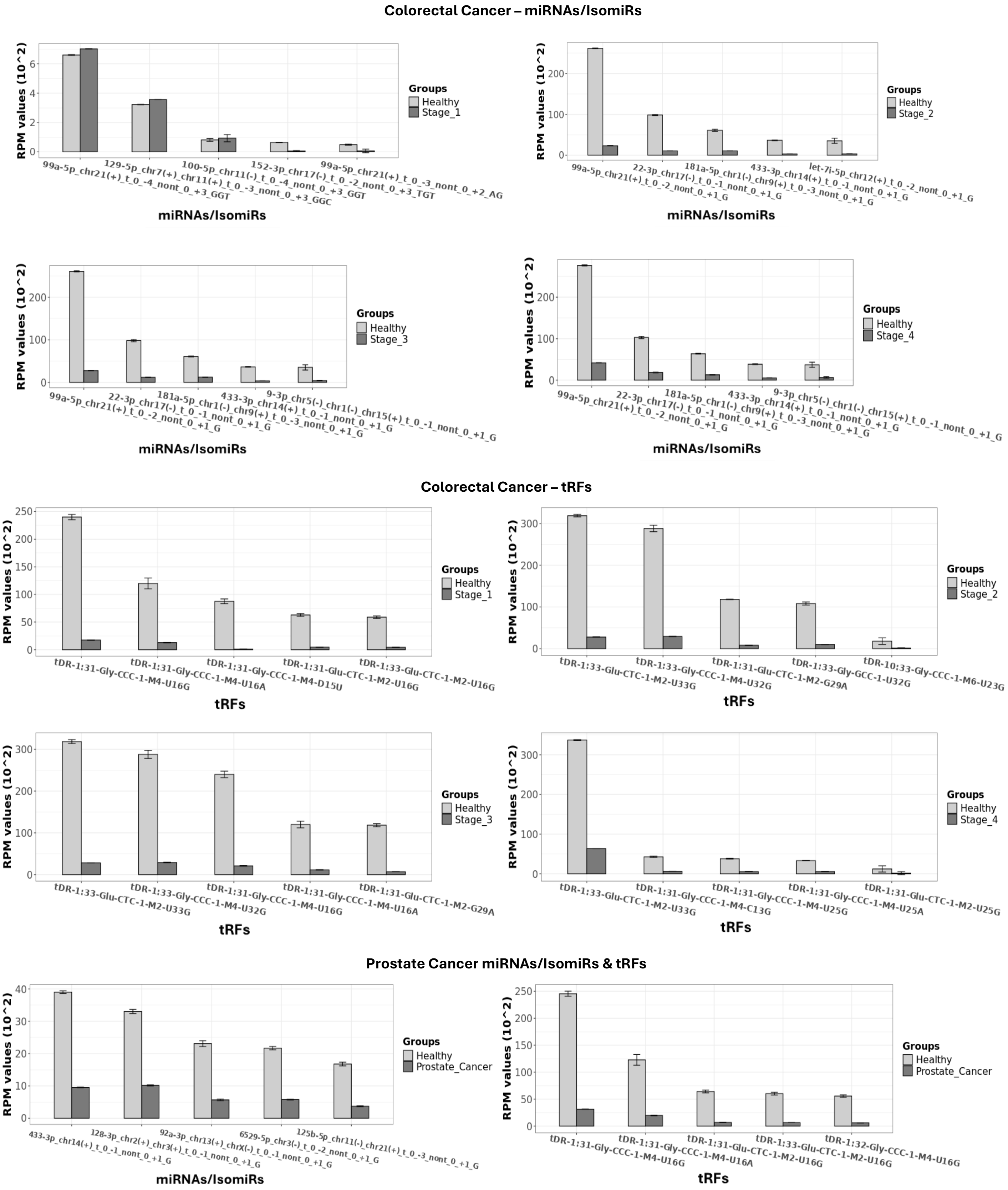

Figure S3. Most abundant significant differentially expressed miRNAs/IsomiRs and tRFs per condition.

.
